# An Efficient Method for the Delivery of the Interleukin-2 Gene to Human Hematopoietic Cells using the  Fiber-Modified Recombinant Adenovirus

**Published:** 2011

**Authors:** V.N. Rogozhin, D.Yu. Logunov, D.V. Shchebliakov, M.M. Shmarov, E.E. Khodunova, I.V. Galtseva, R.V. Belousova, B.S. Naroditsky, A.L. Gintsburg

**Affiliations:** Gamaleya Research Institute of Epidemiology and Microbiology, Ministry of Health and Social Development of the Russian Federation; Moscow state academy of veterinary medicine and biotechnology named K.I. Skryabin; National Research Center for Hematology, Ministry of Health and Social Development of the Russian Federation

**Keywords:** adenovial vector, pseudotyping, interleukin-2, CD46, capsid modification

## Abstract

Recombinant human adenovirus serotype 5 (Ad5/35F-IL2) with modified fibres containing the C-terminal domain fiber-knob of human adenovirus serotype 35, carrying the gene of recombinant human IL-2, has been designed. As a result of the fiber modification, the adenovirus can efficiently deliver the genetic information to bone marrow leukocytes and the tumor blood cells KG-1A (human myeloblastic leukemia cells) and U937 (human histiocytic lymphoma cells), which are normally resistant to Ad5 infection. The flow cytometry data reveal that the modified Ad5/35F penetrates into a population of monocytes, granulocytes, and blast cells of human bone marrow. The expression of interleukin-2 in CAR-negative bone marrow leukocytes (3682.52 ± 134.21 pg/ml) and the cell lines KG-1A (748.3 ± 32.8 pg/ml) and U937 (421.5 ± 59.4 pg/ml) transduced with adenovirus Ad5/35F-IL2 is demonstrated. The fiber-modified adenovirus can be used as a vector for the efficient gene delivery of interleukin-2 to human normal and tumor hematopoietic cells.

## INTRODUCTION

Among the vectors most commonly used to deliver genes to human and mammal cells are vectors based on the human adenovirus serotype 5 (Ad5). The advantages of these vectors are numerous; they are capable of transducing both dividing and non-dividing cells [1, 2]; the adenoviral DNA is not incorporated into the host cell genome and retains its extrachromosomal form; adenoviruses can be produced at a titer of over 10 ^10^ pfu/ml, which enables them to be used as living recombinant vaccines; and they ensure a high expression level of the target gene in a target cell. One of the drawbacks of Ad5-based vectors is their low transducing activity with respect to CAR-deficient and CAR-negative cells. Among those, hematopoeitic cells occupy a significant place. This problem derives from the fact that binding of the adenovirus capsid protein (fiber) and the membrane cell receptor CAR (coxsakievirus-adenovirus receptor) is necessary for the primary interaction between Ad5 and a cell. Therefore, a deficiency or the total absence of these receptors on the cell surface is a factor that limits efficient gene delivery using Ad-5 based adenovectors.

This problem can be overcome via the genetic modification of the adenovirus’ fibers. The modification strategy consists in pseudotyping, i.e., the substitution of fibers or their individual domains for the fibers or similar domains of the adenoviruses of other serotypes, which use receptors other than CAR receptors for binding to the cell surface. This approach was first used in 1996 by Gall *et al* ; they constructed the Ad5 expressing Ad7 fiber and showed the change in vector tropism [3]. Krasnykh *et al* . [4] and Stevenson *et al* . [5] described the production of chimeric Ad5 vectors containing the N-terminal and central domains of Ad5 fiber and the C-terminal domain of Ad3 fiber. Substitution of Ad5 fiber for Ad35 fiber (Ad5/35F) was proposed for the efficient transduction of hematopoietic cells [6]. The modification is made possible by the fact that Ad5 and Ad35 use different receptors in order to bind to the cell surface. It is CAR for Ad5 and the CD46 molecule for Ad35 [7]; a high expression level of CD46 was detected on the surface of hematopoietic cells. These chimeric adenoviruses can efficiently transduce human hematopoietic [6] and dendritic cells [8].

 The described modification of the Ad5 fiber was used to construct a vector capable of efficiently penetrating CAR-deficient hematopoietic cells, with the purpose of delivering the human interleukin-2 gene. The construction of this vector broadens the potential of using vectors based on human capsid-modified adenoviruses, in particular for genetic therapy in the treatment of leukemia of different etiologies.

## EXPERIMENTAL

**Plasmid vectors**

The pZ35 plasmid containing the Ad5 genome with the Ad35 fiber knob domain and shuttle vector pShuttle-CMV-IL2 containing the human interleukin-2 gene regulated by the human citomegalovirus promoter were previously constructed in the Gamaleya Research Institute of Epidemiology and Microbiology [9]; pShuttle-CMV-EGFP was purchased from Stratagene (United States).

**Viruses and bacterial strains**

Recombinant Ad5 expressing a green fluorescent protein reporter gene (Ad5-EGFP), recombinant Ad5 expressing the human interleukin-2 gene (Ad5-IL2) obtained earlier in the Gamaleya Research Institute of Epidemiology and Microbiology [9, 10], Eshcerichia coli strains DH5α and BJ5183.

**Cell lines**

In this study, the following transplantable human cell lines were used: HEK-293 (embryonic kidney cells transformed by the E1 region of the Ad5 genome), KG-1A (myeloid leukemia cells), U937 (monocytic leukemia cells), and the primary leukocyte culture from red bone marrow (RBM) obtained from a healthy volunteer.

**Obtainment of recombinant adenoviruses**

Plasmid constructions carrying the full size adenovirus genome with the modified fiber (Ad5/35F) and the target gene within the genome were obtained via homologous recombination in *E. coli * BJ5183. To this end, *E. coli* cells were co-transformed with a plasmid pair (pZ35 and pShuttle-CMV-EGFP) in order to obtain the plasmid with a full-size genome Ad5/35F and with green fluorescent protein gene (pAd5/35F-EGFP), and pZ35 and pShuttle-CMV-IL2 to obtain the plasmid with full-size genome of Ad5/35F with the human interleukin-2 gene (pAd5/35F-IL2). Prior to the transformation, pZ35 and shuttle vectors (pShuttle-CMV-EGFP and pShuttle-CMV-IL2) were linearized by PacI and PmeI, respectively. In the shuttle vectors, the target genes ( *EGFP * and *IL2* ) were regulated by the human cytomegalovirus promoter. Recombinant clones were analyzed using the polymerase chain reaction (PCR) and restriction assay. Recombinant modified vectors Ad5/35F-EGFP and Ad5/35F-IL2 were obtained via the lipofection of plasmid constructions (pAd5/35F-EGFP and pAd5/35F-IL2) into the HEK-293 line cells using Metafectene Pro agent (Biontex, Germany) according to the enclosed protocol. Recombinant adenoviruses were accumulated in HEK-293 cells and detected on the basis of the development of the characteristic cytopathic effect. The presence of the target genes and fiber modification was proven by PCR and restriction assay. Recombinant modified adenoviruses were purified and concentrated by cesium chloride density gradient ultracentrifugation of the lysates of infected cells. The concentration of adenovirus in the purified specimen was determined spectrophotometrically (λ = 260 nm) using the conversion coefficient 1 OD = 1.12 × 10 ^12^ viral particles/ml. The titer of adenoviral specimens was determined by plaque formation assay on HEK-293 cells.

**Determination of the physical stability of modified adenoviruses**

The physical stability of modified adenoviruses was assessed using the Ad5/35F-EGFP model by determining its thermal stability, according to the procedure described earlier [11].

**Transduction of KG-1A and U937 human tumor blood cells with recombinant adenoviruses**

KG-1A and U937 cells were seeded into 48-well plates at a concentration of 5 × 10 ^4^ cells per well. The cells from the leukocytic fraction of human RBM obtained from a healthy donor were extracted by double centrifugation in the density gradient of the Histopaque-1083 commercial preparation (Sigma-Aldrich, Germany) according to the enclosed protocol and seeded into 48-well plates at a concentration of 5 × 10 ^4^ cells per well. On the following day, the cells were infected with the recombinant modified adenoviruses Ad5/35F-EGFP and Ad5/35F-IL2 and the control adenoviruses with fibers of the wild-type virus and analogous target genes *Ad5-EGFP* and *Ad5-IL2* , at doses of 5 × 10 ^3^ , 10 ^4^ , and 5 × 10 ^4 ^ viral particles per cell.

**Analysis of the efficiency of penetration of adenoviruses into the cells and the expression level of the target gene**

**Table 1:** Concentration and titer of recombinant adenoviruses

Virus	Concentration, viral particles/ml	Titer, pfu/ml
Ad5-EGFP	(4.82 ± 0.17) × 10^12^	(3.80 ± 0.83) × 10^10^
Ad5/35F-EGFP	(2.54 ± 0.21) × 10^12^	(1.22 ± 0.34) × 10^10^
Ad5-IL2	(6.97 ± 0.19) × 10^12^	(5.85 ± 0.76) × 10^10^
Ad5/35F-IL2	(4.23 ± 0.17) × 10^12^	(2.12 ± 0.26) × 10^10^

**Fig. 1:**
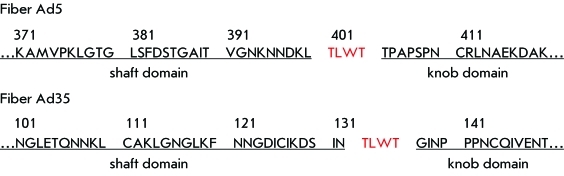
Amino acid sequences of the C-terminal regions of fibers of human avenoviruses serotypes 5 and 35.

The efficiency of the penetration of the recombinant adenoviruses Ad5-EGFP and Ad/35F-EGFP into cells was assessed 48 h post infection via fluorescent microscopy of the infected cells using the EGFP fluorescence intensity.

The expression level of the human IL2 gene in the cells infected with Ad5-IL2 and Ad5/35F-IL2 was assessed 48 h post infection by determining the IL2 concentration in the culture medium of the infected cells by flow cytofluorimetry using the FlowCytomix Bender Medsystems kit (Austria) according to the enclosed protocol. IL2 concentration in the specimens was calculated using the FlowCytomix Pro 2.2 software.

**Determination of leukocyte populations of human RBM which are efficiently transduced by the modified adenovirus**

The cells were infected with Ad5/35F-EGFP at a dose of 10 ^4^  viral particles per cell, in order to determine the leukocyte populations of human RBM into which the modified adenovirus is capable of penetrating. Leukocyte populations in RBM, namely monocytes, granulocytes, T- and B-lymphocytes, and blast cells, were determined 24 h post infection via flow cytofluorimetry, using fluorescently labelled antibodies against CD45 (common leukocyte antigen). The virus-infected cells were detected by EGFP fluorescence and correlated with the corresponding leukocyte population.

## RESULTS AND DISCUSSION

**Obtaining fiber-modified human recombinant adenoviruses serotype 5**

The first strategy for obtaining the recombinant adenovirus Ad5/35F described by Shayakhmetov *et al.* [6] consisted in the substitution of the central (shaft) and the C-terminal (knob) Ad5 fiber domains for the analogous domains of the Ad35 fiber. In order to construct the modified virus, we used the new technology based on a single substitution of the C-terminal knob domain of the Ad5 fiber for the knob fibre domain [7]. The C-terminal knob and central shaft domains of the adenovirus fiber are linked by a short amino acid sequence, ensuring the relative mobility of the fiber knob domain. Most human adenoviruses contain a conservative threonine–leucine–tryptophan–threonine (TLWT) sequence in the mobile region between these domains [12]. The N-terminus of the knob domain of the Ad5 and Ad35 fibers also contains this sequence ( *Fig. 1* ).

**Fig. 2:**
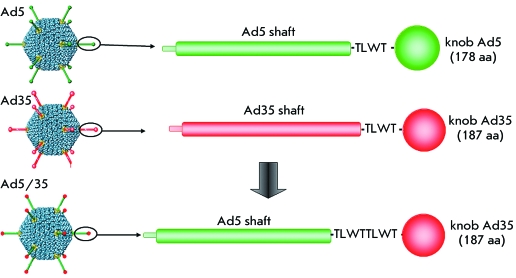
Scheme for modification of fibers of human adenovirus serotype 5 by substitution of its C-terminal knob domain for the same domain of the fiber of human adenovirus serotype 35.

Thus, in order to broaden the tropism of the virus relative to CD46-positive cells, and to achieve proper linking of the shaft and knob fibre domains, we substituted the knob domain of the Ad5 fiber (178 aa) for the knob domain of the Ad35 fiber (187 aa) after the TLWT sequence ( *Fig. 2* ).

**Characterization of recombinant modified adenoviruses Ad5/35F**

**Fig. 3:**
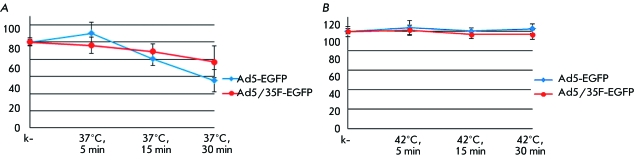
Thermal stability of fiber-modified adenovirions (Ad5/35F-EGFP) as compared with the unmodified adenovirions (Ad5-EGFP). *A* – Heating of adenovirus samples at +37 ^o^ C for 5, 15, and 30 min. B – Heating of adenovirus samples at +42 ^o^ C for 5, 15, and 30 min. Along the X axis, the heating conditions; along the Y axis, the relative efficiency of adenovirus penetration into HEK-293 cells assessed by EGFP fluorescence, %.

Recombinant modified adenoviruses Ad5/35-EGFP and Ad5/35F-IL2 were characterized according to the following parameters: the concentration of viral particles in a sample, titer of the adenoviral sample ( *Table* ), and the physical stability of adenoviruses upon heating ( *Fig. 3* ). Unmodified Ad5 samples containing expression cassettes with EGFP (Ad5-EGFP) and human IL2 (Ad5-IL2) genes were used as a control. As can be seen from the *Table* , concentrations and titers of the samples of modified adenoviruses are comparable with the same parameters of the control samples of unmodified adenoviruses. Thus, the modification of the Ad5/35F adenovirus fiber had no considerable effect on its reproduction.

Since the modification of the adenovirus fibers introduced by us could have some impact on the physical stability of adenoviruses, we assessed the stability of Ad5/35F-EGFP upon heating to +37 and +42 ^o^ C during various time periods as compared with that of unmodified Ad5-EGFP. The thermal stability of Ad5/35F-EGFP virions was shown to be comparable with that of the unmodified Ad5-EGFP adenovirus. Heating the adenovirus samples for 30 min at +37 ^o^ C did not result in a significant decrease in their infectivity ( *Fig. 3A* ). The incubation of adenoviruses at +42 ^o^ C led to the inactivation of adenovirions in both samples: by 11–19% after heating for 15 min and by 23–45% after heating for 30 min ( *Fig. 3B* ).

The data on the thermal stability of the Ad5/35F-EGFP adenovirus attest to the fact that this modification of adenovirus fibers has no considerable effect on its physical stability.

**Assessment of the efficiency of the delivery of genetic information by Ad5/35F-EGFP and Ad5/35F-IL2 adenoviruses to human hematopoietic tumor cells (KG-1A and U937 cell lines)**

**Fig. 4:**
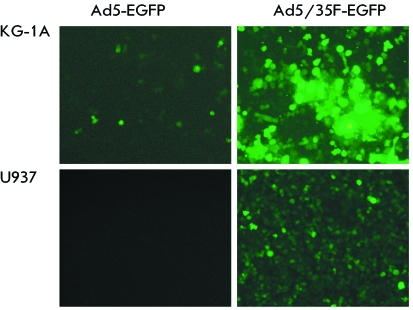
Penetration efficiency of the modified Ad5/35-EGFP virus into KG-1A and U937 lines of human tumor blood cells 48 h after infection.

**Fig. 5:**
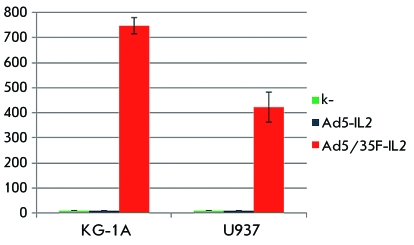
Expression level of the human interleukin-2 gene in KG-1A and U937 lines of human tumor blood cells transduced with recombinant adenoviruses 48 h after infection. Along the X axis, interleukin-2 concentration in the culture medium of infected cells, pg/ml.

We selected KG-1A lines (myeloid leukemia cells) and U937 (monocytic leukemia cells) with a low level of expression of the primary adenoviral receptor CAR [13] and high level of CD46 expression [14], in order to assess the efficiency of *in vitro* penetration of Ad5/35F-EGFP and Ad5/35F-IL2 into human blood tumor cells, and to determine the expression level of the target genes in the virus-transduced cells. The multiplicity of the infection was 5 × 10 ^3^ , 10 ^4^ , and 5 × 10 ^4^ viral particles per cell. The efficiency of the penetration of Ad5/35F-EGFP into the cells was assessed according to the amount of cells expressing the reporter gene *EGFP* and fluorescing under UV irradiation (λ = 395 nm). It was demonstrated that as a result of fibre modification, the modified Ad5/35F-EGFP penetrated into U937 and KG-1A hematopoietic tumor cells more efficiently in comparison with the unmodified virus ( *Fig. 4* ). These results are consistent with the data obtained by other researchers who studied the efficiency of penetration of the modified Ad5/35F-EGFP virus into other lines of blood tumor cells [6] and into primary lymphoid and myeloid leukemia cells [15].

The efficiency of expression of the human interleukin-2 gene in the cell lines transduced with the recombinant modified Ad5/35-IL2 adenovirus turned out to be considerably higher than that in the cells transduced with unmodified Ad5-IL2 ( *Fig. 5* ). The highest concentration of IL2 was noted in the cultural medium of the KG-1A cell line (748.3 ± 32.8 pg/ml) infected with Ad5/35F-IL2. U937 cells transduced with the modified adenovirus expressed the *IL2* gene at a level of 421.5 ± 59.4 pg/ml. In the cells transduced with the unmodified Ad5-IL2 virus, trace amounts of IL2 were expressed.

These results are consistent with the data on the level of CAR and CD46 molecules, the primary receptors for Ad5 and Ad35 adhesion on the surface of the studied cell lines. Hematopoietic cells, including KG-1A and U937 cells, are known to be CAR-negative [13]. Therefore, it is quite understandable that the unmodified Ad5 virtually did not penetrate into these cells; expression of the target genes in them was detected at a trace level. Meanwhile, due to a high level of CD46 expression on the surface of KG-1A and U937 cells [14], the modified Ad5/35F had an enhanced ability to penetrate into these cells, which ensured a high expression level of the target genes in them.

**Assessment of the efficiency in delivering the genetic information to normal leukocytes of human red bone marrow (RBM) by recombinant modified adenoviruses**

**Fig. 6:**
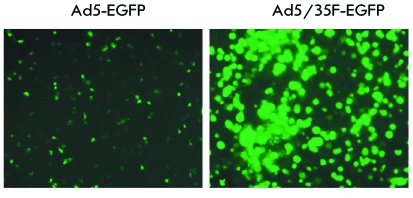
Penetration efficiency of the modified Ad5/35-EGFP virus into leukocytes of human RBM 48 h after infection.

**Fig. 7:**
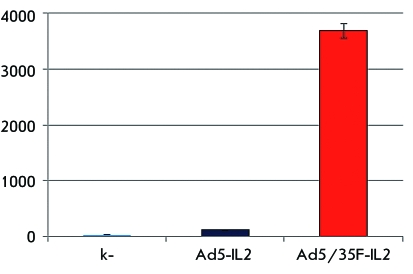
The level of interleukin-2 expression in leukocytes of human RBM transduced with recombinant adenoviruses, 48 h after infection.

The modified Ad5/35F adenovirus efficiently transduces CAR-negative tumor blood cells and ensures the expression of the target genes introduced into its genome in these cells. It was therefore decided to determine the efficiency in delivering the genetic information via adenovectors (Ad5/35F-EGFP and Ad5/35F-IL2) to the cells of the leukocytic fraction of RBM obtained from a healthy blood donor. The reason for this interest is the potential of using the modified adenovirus as a vector for the efficient selective *ex vivo* delivery of genetic information to hematopoietic and immunocompetent human cells. The leukocytes extracted from a RBM sample taken from a healthy blood donor were transfected with modified (Ad5/35F-EGFP and Ad5/35-IL2) and unmodified (Ad5-EGFP and Ad5-IL2) adenovectors at different doses. As a result, the modified Ad5/35F-EGFP vector was shown to penetrate into RBM leukocytes more efficiently in comparison with Ad5-EGFP, which was indicated by the intense fluorescence of 80% of all cells resulting from the EGFP expression ( *Fig. 6* ). IL2 concentration upon cell transduction with vectors with the target *IL2* gene in the culture medium of cells infected with the modified Ad5/35-IL2 was equal to 3682.52 ± 134.21 pg/ml; approximately 30-fold higher than the IL2 concentration in the leukocyte culture medium transduced with the unmodified Ad5-IL2 ( *Fig. 7* ).

**Fig. 8:**
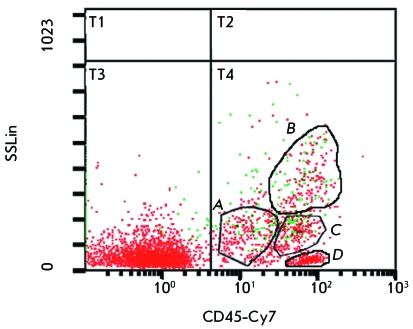
Determination of leukocyte populations in the human RBM efficiently transduced by recombinant modified adenovirus Ad5/35F-EGFP. Adenovirus-infected cells (fluorescing) are shown in green. *A* – blast cells; *B* – granulocytes; *C* – monocytes; and *D* – lymphocytes.

Taking into account the fact that the cells of the leukocyte fraction of the RBM contain a number of heterogeneous cell populations, we decided to determine the populations of leukocytes into which the modified adenovirus efficiently penetrates.

RBM leukocytes were infected with the modified Ad5/35F-EGFP at a dose of 5 × 10 ^4 ^ viral particles per cell. The populations of cells efficiently transduced by the modified adenovirus were revealed 24 h after infection by flow cytofluorimetry using fluorescently labelled antibodies against the common leukocyte antigen CD45 according to the cell size, the expression level of CD45 antigen, and fluorescence of the infected cells resulting from the expression of the *EGFP* reporter gene ( *Fig. 8* ). As expected, Ad5/35F-EGFP easily penetrated into RBM monocytes (32.66% of EGFP-positive cells), which confirms the data obtained earlier [16], and transduced granulocytes (42.86% of EGFP-positive cells) and blast cells (27.24% of EGFP-positive cells), which was first demonstrated. Meanwhile, efficiency in penetration of the modified Ad5/35F-EGFP adenovirus in the subpopulation of T- and B-lymphocytes from human RBM was low (0.71% of EGFP-positive cells). The data on the efficient transduction of monocyte, granulocyte, and blast cell populations with the modified Ad5/35F-EGFP adenovirus are consistent with reports of a high level of CD46 expression in populations of these cells [17]. However, notwithstanding the data on the level of transcription of the *CD46* gene in T- and B-lymphocytes which is comparable to that in cells of the myeloid differentiation branch [17, 18], the modified adenovirus did not efficiently penetrate into lymphocytes. This could be due to the specific regulation of *CD46* expression at the level of the protein.

## CONCLUSIONS

A specific modification consisting in the substitution of the C-terminal knob domain of the Ad5 fiber for the analogous domain of the Ad35 fiber was used to obtain recombinant Ad5 containing the modified fiber and carrying the human *IL2* gene. A manifold increase in efficiency in the penetration of this vector into cells, as compared with the unmodified vector, was demonstrated on KG-1A and U937 line cultures of human tumor blood cells. A 30-fold increase in efficiency in the penetration of the modified vector in comparison with that for the unmodified vector was first demonstrated in experiments on the transduction of the primary leukocyte culture from RBM taken from a healthy donor, which ensures the expression of the human interleukin-2 gene in them. Along with the efficient transduction of RBM monocytes [16], the modified Ad5/35F was first shown to efficiently transduce granulocytes and blast cells of human RBM, while this vector does not penetrate into T- and B-lymphocyte subpopulations.

Based on the results obtained in this study, it can be concluded that the modified Ad5/35F-IL2 adenovirus obtained can be used as a vector for efficient delivery of the human interleukin-2 gene and for its application in the genetic therapy of different types of leukemia, as well as in the design of genetically engineered vaccines capable of efficiently delivering the antigen genes of different pathogens directly to the immunocompetent cells, including dendritic cells, which are professional antigen-presenting cells [19].

## Abbreviations

Ad: human adenovirus
Ad5: Ad serotype 5
Ad35: Ad serotype 35
Ad5/35F: fiber-modified recombinant Ad5
UV: ultraviolet radiation
RBM: red bone marrow
IL2: human interleukin-2
CAR: coxsackievirus and adenovirus receptor
aa: amino acid residue
pfu: plaque-forming unit

## References

[1] Tang D.C., Jennelle R.S., Shi Z., Garver R.I., Carbone D.P., Loya F., Chang C.H., Curiel D.T. (1997). Hum. Gene Ther..

[2] Tang D.C., Johnston S.A., Carbone D.P. (1994). Cancer Gene Ther..

[3] Gall J., Kass-Eisler A., Leinwand L., Falck-Pedersen E. (1996). J. Virol..

[4] Krasnykh V.N., Mikheeva G.V., Douglas J.T., Curiel D.T. (1996). J. Virol..

[5] Stevenson S.C., Rollence M., Marshall-Neff J., McClelland A. (1997). J. Virol..

[6] Shayakhmetov D.M., Papayannopoulou T., Stamatoyannopoulos G., Lieber A. (2000). J. Virol..

[7] Zhang Y., Bergelson J.M. (2005). J. Virol..

[8] Melief C.J., Offringa R. (2001). J. Immunol..

[9] Shmarov M.M., Cherenova L.V., Shashkova E.V., Logunov D.Iu., Verkhovskaia L.V., Kapitonov A.V., Neugodova G.L., Doronin K.K., Naroditskiĭ B.S. (2002). Mol. Gen. Mikrobiol. Virusol..

[10] Logunov D.Y., Zubkova O.V., Karyagina-Zhulina A.S., Shuvalova E.A., Karpov A.P., Shmarov M.M., Tutykhina I.L., Alyapkina Y.S., Grezina N.M., Zinovieva N.A., Ernst L.K., Gintsburg A.L., Naroditsky B.S. (2007). J. Virol..

[11] Rogozhin V.N., Belousova R.V., Logunov D.Y., Shmarov M.M., Lunin V.G., Naroditsky B.S. (2011). Vet. Meditsina..

[12] Guardado-Calvo P., Llamas-Saiz A.L., Fox G.C., Langlois P., van Raaij M.J. (2007). J. Gen. Virol..

[13] Sakurai F., Mizuguchi H., Yamaguchi T., Hayakawa T. (2003). Mol. Ther..

[14] Gaggar A., Shayakhmetov D.M., Liszewski M.K., Atkinson J.P., Lieber A. (2005). J. Virol..

[15] Nilsson M., Ljungberg J., Richter J., Kiefer T., Magnusson M., Lieber A., Widegren B., Karlsson S., Fan X. (2004). J. Gene Med..

[16] Segerman A., Lindman K., Mei Y.F., Allard A., Wadell G. (2006). Virology..

[17] Christmas S.E., de la Mata Espinosa C.T., Halliday D., Buxton C.A., Cummerson J.A., Johnson P.M. (2006). Immunology..

[18] Wang G., Liszewski M.K., Chan A.C., Atkinson J.P. (2000). J. Immunol..

[19] Wang H., Liu Y., Li Z., Tuve S., Stone D., Kalyushniy O., Shayakhmetov D., Verlinde C.L., Stehle T., McVey J. (2008). J. Virol..

